# A Transdiagnostic Approach to Pain and Emotion

**DOI:** 10.1111/jabr.12007

**Published:** 2013-06-25

**Authors:** Steven J Linton

**Affiliations:** CHAMP (Center for Health and Medical Psychology) Department of Law, Psychology, and Social Work, Örebro University

## Abstract

Emotion and pain are known to be intimately related, but treating co-occurring problems is still in its infancy mainly because we lack a clear theoretical understanding of the underlying mechanisms involved. This lack of understanding is problematic because treatment has proved challenging and co-occurring pain and emotional problems are associated with poor outcome, relapse, and greater sick absenteeism. Transdiagnostics has emerged as one way of focusing on the shared underlying mechanisms that drive comorbid problems. This approach has not been thoroughly examined for pain and emotion. Hence, the purpose of this review is to describe a transdiagnostic approach to pain and emotion and its clinical implications. To this end, the transdiagnostic approach is applied to pain and emotion in a narrative review of the literature. A focus on the function of emotion and pain relative to the context is underscored as a way to understand the relationship better. Avoidance, catastrophic worry, and thought suppression are put forward as three examples of potential transdiagnostic mechanisms that may underlie a co-occurring emotion and pain problem. The approach is readily translated to the clinic where assessment and treatment should focus on identifying transdiagnostic mechanisms. However, additional exploration is needed and therefore suggestions for future research are presented.

## Introduction

It is hard to imagine pain without emotion. Indeed, the suffering associated with a nociceptive stimulus is reflected in the emotional response. These emotional responses are usually helpful in dealing with the pain in an appropriate manner. Sometimes however, emotions actually contribute to the development of more problems which is often the case when chronic pain co-occurs with various emotional problems. For example, earlier research demonstrates that pain is associated with emotions such as anger (Trost, Vangronsveld, Linton, Quartana, & Sullivan, 2012), depression (Bair, Robinson, Katon, & Kroenke, 2003; Linton & Bergbom, 2011), fear (Leeuw et al., 2007; Vlaeyen & Linton, 2012), anxiety (Asmundson, Abramowitz, Richter, & Whedon, 2010; Asmundson & Katz, 2009), and worry (Eccleston & Crombez, 2007). In turn, pain and emotion also share many underlying neurophysiologic entities that may either dampen or amplify signals (Bair et al., 2003; Wiech & Tracey, 2009).

When pain is associated with significant negative emotion, treatment and recovery are impeded. This co-occurrence of pain and emotion is in fact, a good predictor of treatment failure, relapse, sick absenteeism, as well as the development of a chronic problem (Lumley, 2010; Mallen, Peat, Thomas, Dunn, & Croft, 2007; Nicholas, Linton, Watson, & Main, 2011). However, a clinical dilemma has been determining the direction of influence, that is, whether the emotion is driving the pain or vice versa (Asmundson & Katz, 2009; Gatchel, Peng, Peters, Fuchs, & Turk, 2007; Keefe, Lumley, Anderson, Lynch, & Carson, 2001). To complicate matters, other types of relationship are also possible, e.g., a third variable influencing both, or independent coexistence. One challenge then, is a better understanding of how emotions and pain are connected to one another.

One common approach is viewing pain and emotion as separate, comorbid entities. This might imply that one independently impacts on the other. In fact, research to date has typically studied a specified emotion such as fear or anger in relation to pain. Interestingly, this insinuates that pain and emotion are something you “possess” (I feel angry). As a consequence, considerable effort is put into diagnosing the exact emotional problem as well as the pain condition to determine their proper diagnosis and thereby the correct comorbidity. Viewing pain and emotion as separate entities puts the spotlight on how the emotional aspects differ from the pain problem and it sets the stage for treating the emotional and pain features, respectively. In other words, treatment might target the pain or perhaps the emotional problem. An age-old dilemma is determining which treatment should commence first, which is mirrored in discussions of “primary” and “secondary” problems.

Treating pain and depression can illustrate the diagnostic approach. Patients seeking primary care for pain also fulfill the criteria for depression more than 50% of the time (Bair et al., 2003; Linton & Bergbom, 2011). When depression is present, treatment failure and relapse are much more likely (Bair et al., 2003; Linton & Bergbom, 2011). Attempts to tackle this problem have focused on treating one of them (the primary problem) (Bair et al., 2003). For example, the pain may be treated in the belief that the depression will reside when the pain problem improves. However, the results of treating one (e.g., the pain) in the hope that both get better have been disappointing (Bair et al., 2003; Linton & Bergbom, 2011). A few studies have tried separate treatments so as to target both problems. For example, in one study (Kroenke et al., 2009) participants with depression and pain diagnoses were offered a pharmacological treatment known to be helpful for depression, and subsequently a treatment for the pain. While the results showed some additional benefits, there were problems in achieving clinically significant improvements in both depression and pain. Together, these studies underscore that current approaches may view depression and pain as separate problems, but treatments based on this approach are not yet satisfactory.

A transdiagnostic approach offers a possible step forward. Details are provided below, but in a nutshell transdiagnostics stresses similar underlying mechanisms that affect both pain and emotion, rather than focusing on how they are diagnostically *different* (Harvey, Watkins, Mansell, & Shafran, 2004). Identifying such factors and treating them theoretically should render benefits to both problems. However, to date, the transdiagnostic approach has not been examined very extensively for pain and emotional problems.

In this paper, it is argued that the area of emotion and pain would benefit from taking a transdiagnostic approach. Instead of searching for how pain and emotion impact on each other, a transdiagnostic approach that identifies underlying mechanisms would afford greater understanding of the processes involved as well as a way forward for treatment. To this end, this paper presents a background for the transdiagnostic approach incorporating the literature on emotion regulation and pain, and then reviews the evidence on whether similar mechanisms might be related to co-occurring pain and emotional problems. Lastly, some examples are provided of how this approach might stimulate clinically relevant research to shed new light on the relationship between emotion and pain as well as new methods for addressing them in the clinic. Because of the nature of the question, this is a narrative review that draws on the rich developments in emotion, pain, and psychology.

## A Failure to Regulate?

The question of how people manage their pain and negative emotion is an intriguing scientific problem with considerable clinical and theoretical implications that has generated a plethora of research. In addition, the development of persistent pain or emotional disorders can be conceptualized as a problem of poor regulation. In the field of emotions, the search for and description of regulation strategies has resulted in an exponential increase in publications. Emotion regulation is defined as “… the set of automatic and controlled processes involved in the initiation, maintenance, and modification of occurrence, intensity, and duration of feeling states” (Webb et al., 2012 p. 144). Several strategies for regulating emotions have been identified with considerable consensus from the research community (Gross, 2007; Gross & Thompson, 2007; Leahy & Tirch, 2011; Webb et al., 2012). These include at least ten methods for downregulating negative affect such as distraction, venting, cognitive reappraisal, withdrawal, and self-reward as well as several for upregulating positive affect such as gratitude, helping others, and expression of positive affect (Webb et al., 2012). Interestingly, these emotion regulation strategies are similar to the ways people cope with pain. Coping strategies for pain also include such tactics as distraction, cognitive reappraisal, withdrawal/avoidance, and self-reward (Linton, 2005; Main, Sullivan, & Watson, 2007; Van Damme, Crombez, & Eccleston, 2008). The regulation or coping perspective even implies that certain mechanisms may be vital such as attention, where there is an optimal level: too much or too little inadvertently exacerbates the symptom (Hasenbring, Hallner, & Rusu, 2012). Yet, there are significant gaps in our knowledge. For example, we have a shaky understanding of the interrelated mechanisms by which pain and emotion develop into chronic problems, as well as how coping works in the treatment of these processes. Further, when they do co-occur, we do not know why nor do we have a clear idea as to which strategies might be best to address them both. We also lack clear clinical strategies for how to actually implement regulation strategies for both conditions.

Given the vast amount of research on the regulation of emotion and pain, we might suppose that answers to the above questions are in sight. However, current research and clinical practice rarely analyze the mechanisms underlying their co-occurrence. The idea of examining the underlying factors however, is not an entirely new idea. Research into both emotions and pain has been cognizant of the need for knowledge about mechanisms. In the emotion regulation area for example, the Action Control Perspective frames regulation in terms of goal pursuit providing a more in-depth picture of how regulation of emotion occurs (Webb et al., 2012). In psychology, the so-called “third wave” therapies such as Dialectical Behavior Therapy (DBT) (Koerner, 2012) and Acceptance and Commitment Therapy (ACT) (Hayes, Levin, Plumb-Vilardaga, Villatte, & Pistorello, 2011) have underscored the need for looking at mechanisms and have pitched the role of emotions into the pursuit of their function rather than as something to be reduced. While the emotion regulation and third wave have set the stage for examining mechanisms, they have failed to fully address the mechanisms associated with the co-occurrence of emotion and pain. Hence, many questions remain. Therefore, the transdiagnostic approach offers a perspective that might enhance our theoretical and clinical understanding of co-occurring emotional and pain symptoms.

Taken together, there is an increasing consensus that both pain and emotion involve various forms of regulation. Moreover, the types of regulation strategies employed are surprisingly similar. Yet, while earlier work has laid a foundation, there is a particular need to address the co-occurrence of pain and emotion.

## Conceptualizing Emotion and Pain From a Transdiagnostic Approach

A transdiagnostic approach offers a new look at emotion and pain. This alternative approach to understanding pain and emotion views them as a regulatory process that is driven by certain shared mechanisms (Harvey et al., 2004). These regulatory mechanisms are vital because they work over time to drive the development of the problem. Consider that both pain and emotional problems often develop over relatively long time spans which provide ample opportunities for shared mechanisms to work (Ehring & Watkins, 2008; Harvey, 2008; Linton, 2002b, 2004, 2005).

### Transdiagnostics

The transdiagnostic approach took shape as an alternative to “diagnostics” as a way of dealing with the fact that most patients suffer more than one problem. This approach has certain advantages. First, transdiagnostics focuses on commonalities across current diagnostic boundaries. Having more than one symptom simultaneously, i.e., comorbid symptoms, is the rule and not something peculiar to emotion and pain (Harvey et al., 2004). Further, many patients with pain suffer any combination of multiple pain sites, insomnia, anxiety, depression and other emotional symptoms (Asmundson et al., 2010; Bair et al., 2003; Breivik, Collett, Ventafridda, Cohen, & Gallacher, 2006; Linton & MacDonald, 2008). Second, transdiagnostics attempts to understand comorbidity by identifying shared mechanisms that drive both problems. In fact, a central problem in working with syndrome-oriented assessment is that patients usually complain of a variety of symptoms and thus have several diagnoses. A diagnostics approach attempts to define the characteristics separating various syndromes creating checklists, e.g., in the Diagnostic Statistical Manual system. Treatment in turn focuses on each syndrome and there is a challenge to prioritize the order of interventions, e.g., primary and secondary ones. On the other hand, the transdiagnostic approach aims to identify mechanisms that transcend syndromes and thereby inform assessment and treatment (Harvey et al., 2004). In other words, transdiagnostics is about mechanisms that cut across diagnostic boundaries and appears to be applicable to emotion and pain. The central question is which mechanisms might drive both emotion and pain?

In order to examine shared mechanisms, a transdiagnostic approach investigates the *function* that emotions and pain serve rather than just their content. In turn, we may understand the function of an emotion or pain best in relation to the context in which it occurs. Because function and context are vital aspect, the next section elaborates on how they are relevant for pain and emotion.

### Function and Context

In order to understand a mechanism from a transdiagnostic perspective, we may study the function it serves and the context in which it is appropriate. Human behavior is described by the interaction between function and context; it is how we interact with the environment. Function considers why a mechanism exists, that is what value it has. The function of emotion or pain has been underscored in a number of models and approaches (Fordyce, 1976; Gatchel et al., 2007; Hayes et al., 2004; Koole, 2009; Vlaeyen & Linton, 2002). Function is understood when we consider that the same response may have different functions. Crying, for example, is usually thought of as an expression of emotion, but it develops over time (e.g., into more intense crying, or even into laughter) and has a function that is highly dependent on the context. Crying may function to gain sympathy (context: a funeral), or it might function to gain congratulations (context: won a prize), or even treatment (context: doctor’s office, in pain). In this section, we explore the possible function of pain and emotion in a transdiagnostic perspective.

An important function that negative emotion and pain may serve is to motivate adaptation that produces balance, that is, homeostasis. As described above, a peculiar observation is that emotional reactions, like pain, are something we strive to modulate. Certainly, we may engage in a number of strategies in order to maintain emotional balance (Gross & Thompson, 2007; Gyurak, Gross, & Etkin, 2011). To be sure, too much or too little emotion can be problematic. Similarly, when we experience pain, we attempt to *cope* with it by engaging in so-called coping behaviors like distraction or avoidance (Skinner, Edge, Altman, & Sherwood, 2003). Therefore, coping is a basic concept in the treatment, management and everyday reaction to pain (Van Damme et al., 2008). In fact, emotion regulation strategies target the situation, attentional processes, appraisal, or the response itself (Gross, 2007), just as coping strategies for pain do (Van Damme et al., 2008). To recapitulate, there are remarkable similarities in how we regulate pain and negative affect with the aim of maintaining balance. Thus, the homeostatic nature of pain as well as emotion is undeniably a striking, shared feature (Craig, 2003). We might conclude that both emotion and pain involve a *process* to affect homeostasis, which in turn should have survival value.

Both pain and negative emotion appear to have the shared function of reducing an unpleasant condition. Intriguingly, there is a need to highlight negative emotion in this respect, as positive emotion serves other functions, e.g., social bonding and adjustment (Gross, 2007). On the other hand, negative affect and pain are associated with various strategies to reduce them. The variety of pain and emotional regulatory coping strategies serve a common function: to reduce unpleasant pain and emotion that restores homeostasis.

Context is also critical (Skinner, 1965) and refers to the environment in which learning takes place (Klein, 1996); that is, the constellation of cues that are in the background when learning occurs. Thus, it can include subtle stimuli like the passage of time or the details of the setting. The role of context came to the forefront in the psychology of pain when it was shown to play a decisive role in learning. An example is the extinction of a learned response that is associated with a stimulus that signals pain. In experiments, a signal such as a red light occurs before the pain stimulus and a conditioned response (fear) or an operant response (push a button that avoids the painful stimulus). During extinction, the red light is presented without a painful stimulus. The participant learns that the light no longer signals a pending pain stimulus and that pushing the button has no effect. After a number of trials the response declines in frequency and is eventually extinguished. It is noteworthy that extinction has been shown to involve the learning of a new relationship between the stimulus and response (Bouton, 2004). Consequently, the main stimulus has two meanings; namely, the old stimulus—response association (signal for pain), and the new stimulus—response association (signal for no pain). In order to determine which will lead to a positive outcome, the subtle i.e., contextual, cues are necessary (Bouton, 2004) such as where (what room, place) the signal occurs. A phobic may learn that a needle is a signal for pain and disgust, but only when they themselves have sought health care, and not when seeing one in a museum. In fact, anxiety is provoked if the contextual cues are not entirely clear since the situation then is ambiguous.

As the reasoning goes then, context is a vital signal for whether a response will lead to a positive outcome (“reinforcement”) or a negative one (e.g., pain). Determining whether a given response serves its desired function then, is a matter of the context in which it occurs. Thus, escape and avoidance may be appropriate in some situations such as when you put your hand on a very hot stove, but inappropriate in other situations like when receiving a required injection (Linton & Fruzzetti, 2013).

*Context sensitivity* is defined as the degree to which an emotion or pain response is appropriate to the situation. Emotions help us adapt to a changing internal and external environment and thereby help us to survive. Therefore, emotional reactions may be more or less in tune with the stimuli present and as a result, more or less efficient in meeting the demands placed on us by the environment. The idea of context sensitivity has grown in the emotion regulation literature (Bonanno et al., 2007; Coifman & Bonanno, 2010a, 2010b) where psychopathology has been linked to the inappropriate expression of emotions or “emotion context insensitivity” (Coifman & Bonanno, 2010a, 2010b). In other words, any given emotional response may be helpful in certain situations, but not suitable in others. Psychopathology then is thought to be related to the “inappropriate” use of emotions in relation to the context. In other words, when emotional responses are not in tune with the environment, they may be counterproductive for the situation leading to more stress and a decreased ability to survive. An illustration is laughter. While laughter may build relationships when exhibited when someone tells a joke, it may also result in ostracizing or anger if exhibited at a funeral (Coifman & Bonanno, 2010a). The context is vital for signaling whether a particular response will lead to benefits or not. Indeed, this is another way to conceptualize problems with emotion regulation such as poignantly described in the literature on dialectical behavior therapy (Fruzzetti, 2006; Fruzzetti, Crook, Erikson, Lee, & Worrall, 2009; Koerner, 2012).

Emotions serve a function relative to the context, and this paper suggests that this is true for pain as well. Consider the following example. Anger facilitates adaptation when a goal is blocked and there is a definable, changeable person or thing responsible for the blocking (Coifman & Bonanno, 2010a). In an acute situation, anger mobilizes our resources to affect a change and better achieve our goals. The anger is short-lived and is a clear response toward a “threat.” On the other hand, anger is not helpful when the goal is distant, diffuse, and the thing blocking the goal is not changeable. The result is likely aggression toward people/things in the immediate environment even though they are not able to alter the block and it contributes to a longer-lasting state of emotional distress (Trost et al., 2012). Consequently, the degree of context sensitivity for an emotion is how well the emotional response functions in the context, ultimately contributing to maintain homeostasis. Similarly, we might learn more about pain by considering how appropriate a pain-related response is in a given context. Our response when in pain can be functional and increase the likelihood of achieving homeostasis. Certainly, pain responses can also be less appropriate. Avoiding bending to lift an object may be adaptive for a person with back pain, if the object is very heavy and the injury is acute. It can also be inappropriate to the context, for example, if the object is light, and the purpose is to avoid (Vlaeyen & Linton, 2012).

Consider a more complex response building upon the example of anger above. Pain can block goals which likely results in anger in the immediate timeframe. This pain-anger response would have value in certain contexts, e.g., when applying for medical care for the first time, since it could be helpful in gaining attention to the injury and prompting timely treatment. The response is functional in gaining treatment and removing a block to life goals. However, in other contexts this response is not helpful.

Let us examine how context insensitive pain-anger responses might create problems. As an example, displaying a pain-anger response at work or with friends, especially long after the injury, may decrease the desired function. This response could have several dysfunctional aspects. First, the pain-anger response reinforces the sufferer’s focus on the pain rather than on pursuing the “blocked” goals. To be sure, the pain-anger response in the longer term is related to poorer coping and more dysfunction (Trost et al., 2012). A steady focus on the pain may reduce problem-solving ability since it restricts “viable” solutions only to removing the pain rather than achieving relevant life goals (Eccleston & Crombez, 2007; Schrooten, Vlaeyen, & Morley, 2012; Van Damme et al., 2008). Second, the pain-anger response described is deleterious to building cooperation with others, e.g. health care professionals or family, since such a response fosters cognitive beliefs of entitlement (Cano, Leong, Heller, & Lutz, 2009) and more negative affect associated with feelings of injustice (Sullivan et al., 2008). The ensuing communication of “it’s someone else’s fault” and “I have the right” results in others taking distance. In an experimental situation, for example, people viewing videos of angry people in pain rated them as less desirable and more difficult to communicate with (Burns et al., 2012). Finally, the response can lower the patient’s engagement for treatment because of the focus on removing the pain. Consequently, a pain-anger response that is not context sensitive may be a driver in the development of persistent pain problems (Trost et al., 2012), rather than serving the function of receiving appropriate help and pursuing life goals. It might also drive the emotional problem.

Context insensitive pain-anger responses are also congruent with current conceptualizations concerning goal pursuits. Theoretically, when there is a threat to a personal goal, this threat may be handled by accommodation (attempt to adjust desires and preferences) or assimilation (actively attempt to change situation), where accommodation results in flexible goal adjustment and assimilation in persistent goal pursuit (Schmitz, Saile, & Nilges, 1996; Schrooten et al., 2012; Van Damme, Legrain, Vogt, & Crombez, 2010). Emotional or pain responses that are insensitive to the context appear to mirror the assimilation process and lead to tenacious goal pursuit.

Interestingly, we cast emotion and pain in a new light when we consider their function in relation to the context. The relationship may better capture the intricate interaction between how we react when experiencing pain and emotion and the environment. An overriding function of emotion and pain is as “motivators” or drivers to maintain homeostasis. Negative affect and pain are typical threats to well-being and thus motivate action to reduce them. Various mechanisms for reducing discomforting emotion/pain then are transdiagnostic in nature. What kinds of mechanisms might work in this transdiagnostic way?

### Transdiagnostic Mechanisms that may Serve Emotion and Pain

In this section, we will consider three examples of mechanisms that might operate in a transdiagnostic fashion for co-occurring emotion-pain, from the perspective of function in relation to context, to observe how this view might aid our understanding.

#### Avoidance

Avoidance is no stranger to either emotion or pain. Avoidance is based on learning where previous experience with an aversive (emotional or painful) stimulus creates “threat” cues. In turn, when threat cues are present, certain behaviors occur in order to avoid the aversive event. A tone that precedes a painful electric shock quickly takes on threat value and cues avoidance responses. Similarly, we can avoid situations that result in negative affect. Here again cues, e.g., seeing a certain person approaching (that has previously resulted in an angry argument) becomes a threat cue where we may avoid by walking the other direction. For pain as well as for anxiety disorders such as phobias, posttraumatic stress disorder, obsessive-compulsive disorder, and panic avoidance models are the state-of-the-art for understanding them (Asmundson et al., 2010; Barlow, 2004; Farmer & Chapman, 2008; Leeuw et al., 2007; Vlaeyen & Linton, 2012; Vlaeyen, Morely, Linton, Boersma, & de Jong, 2012). Avoidance then is one mechanism that appears to contribute to homeostasis since it helps to reduce immediate and high levels of negative affect or pain.

Avoidance has also been underscored in third-wave conceptualizations of the development of problems. For ACT, experiential avoidance plays a central role in psychopathology and has to do with efforts to avoid or escape from aversive private events such as emotions or bodily sensations (e.g., pain) (Valdivia-Salas, Sheppard, & Forsyth, 2010). Experiential avoidance is considered as one part of emotion regulation (Valdivia-Salas et al., 2010) and in fact, experiential avoidance was underscored as a sort of transdiagnostic factor more than a decade ago (Hayes, Wilson, Gifford, Follette, & Strosahl, 1996). Likewise, avoidance of emotions and bodily sensations is central in DBT where such avoidance may subsequently result in dysregulation of emotional responses and the development of problems (Koerner, 2012). Therefore, the idea that avoidance may be an underlying mechanism is congruent with the developing third-wave database.

While avoidance can reduce aversive private events, it may paradoxically lead to an escalation of the emotional or pain problem. When avoidance crosses the border from maintaining homeostasis to so-called experiential avoidance, it may actually increase the problem (McCracken, 2006; Rachman, 1998; Vlaeyen & Linton, 2012). When the avoidance functions to decrease actual threat, it has true value. However, as the threat stimulus generalizes to other stimuli and avoidance becomes predominant, it may be the fear itself that is being avoided rather than a true threat. This is one explanation for the development of problematic avoidance. Avoidance then can be helpful when it functions to reduce actual threat in a given context. It can also be a driver of the chronification process if the avoidance is context insensitive.

#### Catastrophic worry

In the field of pain, catastrophizing is a key player in several models such as the fear-avoidance model (Vlaeyen & Linton, 2000) and the misdirected problem solving model (Eccleston & Crombez, 2007). Similarly, a key to emotional dysregulation in several models of psychopathology is repetitive negative thinking (Ehring & Watkins, 2008; Watkins, 2008). Indeed, catastrophizing and repetitive negative thinking are a process where thoughts, emotions and overt behavior are intertwined (Flink, Boersma, & Linton, in press). We have argued that catastrophizing might be conceptualized as a form of *repetitive negative thinking*, similar to worry or rumination (Flink et al., in press). Consider that repetitive negative thinking has been defined as “a style of thinking about one’s problems (current, past, or future) or negative experiences (past or anticipated) that is *repetitive*, at least partly *intrusive*, and is *difficult to disengage* from” (Ehring et al., 2011). Evidently, this broad definition serves for worry and rumination as well as for catastrophizing. To emphasize the similarities between these concepts, we put forward the term *catastrophic worry* (Flink et al., in press).

This line of reasoning is inspired by the growing research about repetitive negative thinking as a *transdiagnostic* construct, identified across disorders (for a review, see Watkins, 2008). According to this research, there are more similarities than differences between different forms of repetitive negative thinking such as worry, catastrophizing and rumination. As an example, rumination is “a passive focus on one’s symptoms of distress and the possible causes and consequences of these symptoms. The individual repeatedly goes over problems and his or her feelings about the problems, without moving into constructive problem solving” (Nolem-Hoeksema, 2005). This definition serves well for catastrophic worry.

Catastrophic worry may serve a similar function for emotion and pain. The intrinsic *function* of catastrophic worry apparently is to reduce negative affect that might arise in a stressful situation such as suffering from persistent pain or emotional distress (Flink et al., in press). As with avoidance responses above, catastrophic worry is appropriate in certain contexts, but not in others. If you are walking home late at night on a dark street and hear footsteps in the dark it is appropriate to entertain catastrophic worry. This will help to prepare for possible emergencies such as an attack or someone who is in need of help. Catastrophic worry however, becomes a transdiagnostic driver of problems when it occurs in other contexts (I may need to walk home someday, or walking on a busy street during the day) and spins out of control. While context insensitive, the function of the catastrophizing might be to reduce other, even more negative, emotion. The negative thoughts and feelings being avoided are much “worse” and often deal with intense and severe situations such as going insane or death.

Catastrophic worry then is an example of a possible transdiagnostic process that may serve as a driver of emotion and pain problems.

#### Suppression

A final example is suppression as an active attempt simply to keep the emotion or pain out of mind (Magee, Harden, & Teachman, 2012). It is an active strategy involving the inhibition of the emotion or pain. The likely function of suppression is to reduce undesirable thoughts and associated emotion. As with avoidance and catastrophic worry, suppression paradoxically may maintain the problem. Put simply, in order to hold the emotion or pain in check, one needs to constantly check and control for its occurrence, which in itself increases the undesired emotion or pain (Watkins & Moulds, 2009). This is the basis of the infamous “white bear experiment” where participants are firmly instructed *not* to think about a white bear. Paradoxically, the typical result is that a white bear quickly comes to mind (Wegner, Schneider, Carter, & White, 1987). Suppression is appropriate in certain situations, e.g., a crisis. In order to respond to a crisis, emotion or pain may need to be suppressed so that we are not overwhelmed by it, thus allowing us to deal with the situation. However, when we begin to constantly check and control emotions or pain, even in non-threatening situations, the threat cues may become overwhelming so that we attempt to suppress them entirely. The expected result would be a dramatic increase in suffering (Hasenbring et al., 2012; Hasenbring & Verbunt, 2010). Consequently, suppression may function on the short term to reduce unwanted thoughts and emotion or pain, but when employed in a context insensitive manner it may increase a pain or emotional problem.

## Translational Discussion

The transdiagnostic view concentrates on shared mechanisms where the function of emotion and pain in context is in focus and it has several clinical implications. Given that both emotion and pain involve remarkably similar regulatory processes, shared mechanisms that function to maintain or restore “homeostatic” balance are plausible. This conceptualization might serve to promote new avenues for understanding the relationship. In addition, if the transdiagnostic approach is of real value, it will translate into more effective treatment. This discussion section then, focuses on issues relevant for translational research.

It is noteworthy that a central key to understanding the transdiagnostic perspective for pain and emotion is considering both the *function* and *context sensitivity* of the proposed mechanism. This helps to explain how strategies can be effective in certain situations, but paradoxically drive the development of the problem when employed out of context. For example, avoidance, catastrophic worry, and suppression strategies may work well to reduce distressing negative states related to an actual threat in acute pain, but instead catalyze the development of both emotion and pain problems when context insensitive.

The transdiagnostic view has many clinical implications that are amenable to testing in the clinic or the laboratory. Table 1 summarizes some of the main implications and areas in need of research.

**Table 1:** An Overview of the Main Implications Derived from a Transdiagnostic Perspective of Emotion and Pain

	Implication	Description	Comment
1. The function of emotion and pain responses is central for understanding how pain and emotion work.	Pain and emotion are part of a regulatory system. Various emotional and pain responses may function to reduce to distressing symptoms. Focus is on function rather than content.	This is in contrast to examining only the content of emotion or pain. Research needed on specific functions.
2. The function of emotion and pain is relative to the context.	The situation in which a trigger for pain or emotion occurs is highly relevant for whether the regulation strategy will be helpful. The same strategy may be appropriate or not appropriate depending on the situation.	Emotion regulation and pain coping strategies have usually been studied in terms of content, i.e., the type of strategy e.g., distraction. Studies of contextual sensitivity are direly needed.
3. Emotion and pain may share important mechanisms.	These mechanisms serve the regulatory function of maintaining or restoring homeostasis that is reducing negative emotion and pain.	These mechanisms are transdiagnostic and are believed to be the drivers in the development of both emotion and pain problems. Studies needed to examine their role in both emotion and pain
4. Several mechanisms are suspected to be relevant transdiagnostic drivers of pain and emotion.	Based on the literature, catastrophic worry, suppression and avoidance behaviors are offered as examples of possible transdiagnostic mechanisms.	These mechanisms are known to be salient for emotion and pain separately. There is a need to study their impact for both pain and emotion over time.
5. A transdiagnostic approach has important clinical implications.	Identifying potent transdiagnostic mechanisms would suggest that treatments that address these would have benefits for both pain and emotion.	It has been difficult to treat both pain and emotional problems. There is a need to develop assessment and treatment methods to be clinically tested.

### Research Implications

Although the transdiagnostic approach is compelling, it is still in its infancy and therefore in need of testing. The conceptualization leads to several testable hypotheses and four are briefly outlined here as examples.

First, we might study the intriguing idea of context insensitivity. We have seen above that a mismatch between an emotion and the context is associated with maladjustment and future psychopathology. I would therefore predict that pain responses that are context insensitive would produce more dysregulation of emotion and pain and thereby contribute to the development of chronicity. Indeed, there is reason to study whether simply expressing pain in inappropriate situations is associated with future problems. First, there is a need to develop and assess methods for determining the degree of context sensitivity for responses to painful stimuli. Subsequently, clinical studies might assess context sensitivity for pain responses and then follow participants over time to evaluate their effects. The hypothesis might also be tested in the laboratory where context appropriate responses are compared to context inappropriate responses for their effect on regulatory processes and outcome variables. Still another interesting test of the idea is to follow the development of context sensitivity. For example, we know too little about how children learn to process and regulate pain stimuli, but this could be studied in longitudinal research.

Second, investigating recovery from a triggered episode of pain or emotion could highlight the mechanisms shared. Emotion and pain are everyday occurrences. The problem may *not* be that they occur, but rather how balance is achieved afterwards. This paper predicts that similar mechanisms would be employed for emotion and pain that function to restore homeostasis. Further, studying recovery might reveal key information for the prevention of chronicity. Recovery from emotional dysfunction, as an illustration, has three different trajectories depending on emotion context sensitivity (Bonanno, 2004). After traumatic events, about 35% to 60% of the sufferers are said to be resilient showing mild disruption of normal functioning and a relatively fast recovery. This is in contrast to another trajectory (15%–35% of sufferers) that has moderate disruptions and where recovery takes more than 12 months. The third trajectory (10%–15% of the sufferers) is chronicity where severe disruptions are experienced and there is little recovery over the course of 12–24 months. These trajectories appear to be similar to the trajectories observed after an episode of back pain where many are resilient and recovered quickly and a smaller number have difficulties recovering (Gatchel et al., 2007; Linton, 2002a, 2005; Waddell, 2004; Waddell, Aylward, & Sawney, 2002). Studying shared mechanisms in longitudinal investigations could shed light on how persistent emotion and pain problems develop. There are lessons to be learned from the literature on emotion that might be shared mechanisms with pain.

Third, the common underlying mechanisms suggested in the model might mean that there are also common modulators of both pain and emotion. These modulators might make certain individuals more susceptible to the underlying mechanisms. For example, factors such as anxiety sensitivity or distress tolerance might make catastrophic worry more likely. If true, this would translate to clinical utility in the early identification of patients at potential risk for developing a problem.

Fourth, the regulatory role of pain and emotion raises the question of the function of positive emotion. The field of positive psychology focuses on the role of positive emotion rather than the usual focus on disturbing negative emotion, not least because the two are not simply mirror images of one another (Hanssen, Peters, Vlaeyen, Meevissen, & Vancleef, 2012; Lee Duckworth, Steen, & Seligman, 2005). However, the role of evoking positive emotion on pain is not yet clear. One investigation manipulated positive emotion (optimism) and found that inducing optimism resulted in lower pain intensity (Hanssen et al., 2012). Interestingly, the effect was mediated by changes in catastrophic worry. Thus, studying the role of positive emotion in situations where negative emotion or pain is triggered may help us to understand the important mechanisms involved in regulation.

### Implications for Clinical Practice

This paper highlights the potential for researching a transdiagnostic approach in the clinic. Three unresolved areas invite application. First, is the question of how patients might best be assessed in order to capture transdiagnostic mechanisms? For example, little is known about which mechanisms are shared, especially for the numerous combinations of emotion and pain that exist. What are the mechanisms? Reviews of the transdiagnostic approach, as well as the emotion, and pain literature provide impetus for us to tease out the relevant mechanisms. Further, is the problem of whether such mechanisms applicable to all emotion and pain? Would the shared mechanisms be different for an anxiety and pain comorbidity (Asmundson & Katz, 2009) as for a depression and pain comorbidity (Linton & Bergbom, 2011)? Does the type of pain matter: would the mechanisms be different if the problem regards back pain or cancer pain? Lastly, we need to study how individual profiles of mechanisms are related to the trajectory of recovery and return to normal function. The transdiagnostic view implies that certain individuals may harbor a set of mechanisms (a profile) that is related to rapid recovery or to the development of long-term disability. Priority is given to this research because of the tragic consequences associated with chronic disability.

Second, if shared mechanisms are identified then treatment that addresses these should result in improvements for pain, emotion and normal functioning. Although comorbid emotion and pain problems are known to be associated with poor treatment outcomes, tackling this problem is challenging. Treating one problem at a time has little evidence to support it, while simultaneous treatments may also be disappointing as suggested above in the example of the attempt to treat both depression and pain (Kroenke et al., 2009) that produced some additional benefits, but did not solve the problem as only 26% of the patients receiving both treatments achieved combined benefits for both depression and pain. This illustrates that there is much to be learned about treatment and underscores the need to test transdiagnostic methods that target shared mechanisms.

Third, the transdiagnostic approach to emotion and pain produces unique ideas for the early, secondary prevention of persistent disability. The approach outlined here predicts that the mechanisms driving the development of persistent disability are present very early on. In fact, response patterns and context sensitivity are hypothesized to take shape during childhood and to continue to be formed during adolescents and adulthood. While pliable, early learning experiences set the stage for development and constitute a pattern of responding. The literature on the development of chronic disabling back pain points out that mechanisms such as pain-related fear and catastrophic worry may be in play within hours of a painful injury (Leeuw et al., 2007; Linton, 2002b). Furthermore, screening procedures that in part tap into these mechanisms have successfully identified patients who likely will development long-term pain-related disability (Hill, Dunn, Main, & Hay, 2010; Maher & Grotle, 2009; Melloh et al., 2009; Nicholas et al., 2011; Reme et al., 2012). In addition, the conceptualization outlined in this paper maintains that a response to a stimulus that triggers pain and/or emotion is modifiable. This predicts that teaching skills to alter the emotion and pain regulation strategies targeting the underlying shared mechanism should have considerable effects. In essence, this would remove potent drivers in the chronification process and prevent the development of persistent disability.

In summary, pain and emotion are remarkably similar and may share important underlying mechanisms that can help people regulate them. A transdiagnostic view of emotions and pain that focuses on function and context opens the door to exciting research that will promote our understanding of these problems, offering promise for better assessment and treatment of patients suffering emotion and pain problems. In fact, the transdiagnostic approach offers the promise of better clinical results since treatment would focus on the shared mechanisms driving both the pain, but also the emotional problem.
